# Everybody's Page

**Published:** 1892-04-23

**Authors:** 


### Everybody's Page.

In America an ordinary drunkard, who has not immortalised himself by having delirium tremens, is called a "plain drunk," if he is brought into hospital frequently as an inebriate, he is dignified with the name of "rounder."

The variation between the night and day measurements of the thermometer is unusually small in Malvern, a fact which contributes in no small degree to the value of the place as a health resort to invalids. Another feature in the climate of the place which must commend itself even to the most robust of Londoners is the absence of fog and mist in the months of November and December, when the inhabitants of this overgrown metropolis are usually shrouded in gloom, darkness, and depression of spirits.

Mr. Edison has done so much for the world in showing us how sound can be conveyed, is it too much to hope that he will invent some simple means for the deadening of sound? The importance of shutting out all noise from sick rooms and from the ears of nervous invalids is only equalled by the difficulties in the way of accomplishing the desired end. There are thousands of sensitive people as well as invalids whose lives are worried by the irritating noises which too often penetrate the thin walls of modern houses replete with every *inconvenience.*

A curious story of blood poisoning comes from Liverpool. The practice of stopping the bleeding of a wound by covering it with a cobweb is familiar to most people. A woman who had cut her hand endeavoured to stop the bleeding in this manner, but soon afterwards blood poisoning set in, and she died. The result of this points a moral. If people use cobwebs, they should be careful that the cobweb, like any other dressing, is free from dirt. It is probable that in this case there was dirt of a poisonous nature on the cobweb used, though no direct evidence seems to have been adduced to prove this.

Lovers of the fruit which so rudely surprised Isaac Newton into the discovery that made him famous will find food for reflection of no very cheerful or reassuring nature in the conflicting evidence brought forward as to the presence of arsenical poisoning in American and Canadian apples. It has just been asserted by an eminent Canadian botanist that an analysis of apples which have been sprayed with Paris green shows them to be wholly untainted with arsenic. An equally eminent authority on this side of the water takes issue with this conclusion, chiefly on the ground that if the spraying is to be effectual the fruit must be affected injuriously, and is firm in his belief that if Canadian fruit is not infected, American is. In whom are we to put our faith?

Little hand-cars, or jinrikishas, take the place of carriages in Japan, and men runners do the work of pulling better, faster, and more untiringly than horses. The women are dressed in a straight, loose garment called a kuciom, made of silk or crape, and of beautiful colours and designs. It is folded across the chest and confined at the waist by a broad, stiff sash or obi, which is tied in an immense bow supported by a bustle behind. Their stockingless feet are thrust into cotton feet-gloves with a division for the big toe, through which passes the strap of the straw sandal or wooden clog used when walking out of doors. The hair, even of the poorest, is elaborately dressed in bows and chignons. The women have the perfect manners of self-respecting restraint; trained to please, they are always cheerful, and taught to control themselves, brawling voices are unheard. A child is never slapped or scolded in Japan. With vanity sufficient to produce neatness of appearance and beauty of dress, the Japanese ladies are spared the vagaries of fashion, and custom does not yet necessitate the wearing of hats, bonnets, flowers, stockings, boots, shoes, petticoats, underlinen, or jewellery, so that simplicity can still characterise the life of women of even the wealthiest families.

